# Autologous Micro-Fragmented Adipose Tissue (MFAT) Injections May Be an Effective Treatment for Advanced Knee Osteoarthritis: A Longitudinal Study

**DOI:** 10.3390/jcm14186571

**Published:** 2025-09-18

**Authors:** Joachim De Groote, Caro Roten, Elizaveta Fomenko, Pascal Coorevits, André Harth, Yves Depaepe

**Affiliations:** 1 Faculty of Medicine, Ghent University, 9000 Ghent, Belgium; 2 Faculty of Medicine, KU Leuven, 3000 Leuven, Belgium; 3 Department of Public Health and Primary Care, Ghent University, 9000 Ghent, Belgium; 4 Department of Orthopedic Surgery, AZ Jan Palfijn, 9000 Ghent, Belgium

**Keywords:** mesenchymal stem cells, regenerative medicine, knee osteoarthritis, adipose-derived stem cells, intra-articular injections

## Abstract

**Background/Objectives**: Knee osteoarthritis (OA) is a major cause of pain and functional disability worldwide, leading to a growing interest in more durable and less invasive therapies. Micro-fragmented adipose tissue (MFAT) injections have emerged as a promising frontier in regenerative therapies using mesenchymal stem cells (MSCs). This study assessed the safety and effectiveness of MFAT injections for symptomatic knee OA while investigating the duration of treatment effects. **Methods**: This longitudinal study screened patients with symptomatic Kellgren-Lawrence (KL) grade II-IV knee OA who received single-dose MFAT injections. Outcomes were assessed using the Knee injury and Osteoarthritis Outcome Score (KOOS) subscales at baseline, 3, 6, and 12 months. A linear mixed effects model was performed to explore how age, BMI, sex, and OA severity influence outcomes. **Results**: Among 39 evaluable patients, mean baseline KOOS was 46.5 (SD 18.1). KOOS scores improved significantly across all subscales, peaking at six months and remaining higher than baseline at 12 months. Improvements exceeded clinically meaningful thresholds, including KL grades IV. Female patients reported significantly worse overall outcomes than male patients (*p* < 0.05). Minor self-limiting synovitis was reported in 18% of cases, and no severe adverse events were observed. **Conclusions**: MFAT infiltration may represent a safe, minimally invasive option to improve symptoms and delay surgery in patients with knee OA, including those with advanced disease. These findings highlight the potential role of MFAT as part of the treatment algorithm for knee OA, although strategies to sustain long-term benefits and confirmatory trials are needed.

## 1. Introduction

Osteoarthritis (OA) affects 3.8% of the global population, primarily impacting weight-bearing joints like the knee and hip, with a higher prevalence among females and older individuals [1,2,3,4,5]. Risk factors include physical inactivity, malalignment, repetitive movements, and obesity [2,3]. OA leads to progressive cartilage and bone degradation, causing synovitis, pain, and stiffness, which hinder daily activities [6,7,8].

First-line therapies, such as lifestyle changes, physical therapy, and non-steroidal anti-inflammatory drugs (NSAIDs), provide temporary symptom relief but do not halt tissue degeneration. At the same time, prolonged NSAID use can cause severe side effects [1,9,10]. Intra-articular injections (e.g., hyaluronic acid or corticosteroids) offer only short-term benefits, and their efficacy is debated [10]. Total knee arthroplasty remains the standard for end-stage OA but is often considered premature for young patients and can result in dissatisfaction in 20% of patients [11]. Less invasive alternatives, like platelet-rich plasma (PRP) and mesenchymal stem cells (MSCs), show promise in slowing OA progression and delaying knee arthroplasty [4,7].

The regenerative and anti-inflammatory potential of MSCs drives the growing interest in this approach. They produce bioactive molecules with immunomodulatory, anti-inflammatory, and angiogenic effects and can differentiate into chondrocytes, promoting tissue regeneration [4,7,9,12]. For autologous transplantation, adipose tissue and bone marrow are the main MSC sources. Adipose tissue is preferred as it is less invasive to harvest and yields higher MSC concentrations. Additionally, viability is influenced by age in the case of bone marrow, a factor that holds relevance for the majority of OA patients [4,11,12,13].

Micro-fragmented adipose tissue (MFAT) has emerged as a promising frontier in regenerative therapies. Several studies have demonstrated MFAT’s effectiveness in pain reduction and functional improvement [4,7,8,9,10,11,13,14,15,16]. This even extends to cases of Kellgren-Lawrence grade IV, a classification typically indicative of the need for knee replacement surgery [5,7].

The response to MFAT treatment varies, with patients in lower KL grades (II–III) generally experiencing more significant and longer-lasting improvements compared to those with KL grade IV, where advanced cartilage degradation and subchondral bone changes may limit the regenerative effects [13,17,18]. However, more advanced grades of OA are often not included in previous studies.

Additionally, MSC regenerative potential tends to decline with age, leading to less favorable outcomes in older patients. Younger individuals tend to show more sustained improvements in pain and function [11,13]. The correlation between BMI and treatment outcome has been discussed in former studies, but no significant correlation has been found [11,13,19].

Moreover, there is still uncertainty concerning the duration of the therapeutic effect. Some studies suggest that it wanes after 12 months [6]. The knowledge gap extends to the safety profile of MFAT treatment. Literature showed that adverse events were rare. The most reported adverse events are either procedure-related or linked to a reaction to MFAT itself, with synovitis being the most common [5,9,13].

This study aims to evaluate the effectiveness and safety of MFAT injections in patients with symptomatic knee OA (Kellgren-Lawrence grade II–IV). We hypothesize that MFAT injections significantly improve pain, function, and quality of life in the short to mid-term, with a favorable safety profile. Additionally, we explore potential differences in treatment response based on sex, age, BMI, and disease severity. These findings could enhance patient selection and refine treatment strategies for this minimally invasive therapy.

## 2. Materials and Methods

### 2.1. Study Design and Population

In this single-center, longitudinal observational study, 58 patients with symptomatic knee OA were treated with MFAT injection between January 2021 and May 2023, and were recruited and screened for eligibility. Informed consent was obtained from all patients, and the Ethics Committee of Jan Palfijn Hospital in Ghent approved the study on 7 November 2024 (Reference number B7132024309).

Eligible patients were 18–70 years old with symptomatic knee OA, including KL grades II–IV, confirmed by radiographs or magnetic resonance imaging (MRI). Symptoms were lasting longer than 3 months, and prior conservative treatments during these 3 months (e.g., Anti-inflammatory drugs, physical therapy, intra-articular injections with hyaluronic acid, corticosteroids, or PRP) were not successful. For practical reasons, comprehension of the Dutch questionnaire was required. Exclusion criteria included intra-articular injections during the last 3 months, uncontrolled systemic or rheumatic diseases, malignancies, and interfering lower limb pathologies.

### 2.2. Procedure for Obtaining Adipose-Derived Mesenchymal Stem Cells

The MFAT procedure was performed under general anesthesia using the MYFILL^®^ device (BioRep, Milan, Italy). MFAT preparation and injection followed a standardized protocol across all patients, using the same device and processing steps. The abdominal donor site was infiltrated with a solution of NaCl (0.9% 250 mL) and adrenaline (1 mg). After 15 min, manual liposuction was performed using a cannula (Ø 1.7 × 2.1 mm–8 holes Ø 1.2 mm) connected to a 10 mL syringe, filling 4–5 syringes in 2 mL increments to preserve adipose cell integrity. The tissue was decanted, and the remaining adipose layer was centrifuged (Adip’spin, Adip’Sculpt, 2 rue Paul Milleret, Besançon, Franche-Comté, France) 3 times with NaCl washes. After centrifugation, 5–10 mL of micro-fragmented adipose tissue (MFAT) was extracted and injected into the osteoarthritic knee joint using an 18-gauge needle. Some patients received unilateral, others bilateral infiltration. For patients with bilateral OA, only the most symptomatic knee was considered for analysis to avoid within-patient clustering. Outcomes were therefore analyzed at the level of the individual, not the joint, ensuring that each patient contributed a single data point. This approach was chosen to reduce heterogeneity and avoid inflating the effective sample size.

Patients were encouraged to continue daily life activities immediately after treatment when tolerated. However, explosive sports were discouraged during the first six weeks. NSAIDs were not recommended either for the first six weeks as this could interfere with the anti-inflammatory effects of MFAT injection.

### 2.3. Outcome Measures

Demographic data, including age, sex assigned at birth, BMI, and Kellgren-Lawrence (KL) grade [20], were collected. For bilateral OA, the most symptomatic knee was assessed to streamline data collection. Clinical outcomes were evaluated using the validated Knee injury and Osteoarthritis Outcome Score (KOOS) questionnaire, consisting of 42 items across 5 subscales: Symptoms (7 items), Pain (9 items), Activities of Daily Living (ADL) (17 items), Sport and Recreation (5 items), and Quality of Life (QoL) (4 items), scored on a 5-point Likert scale [21,22,23]. The KOOS questionnaires were self-administered, either electronically or on paper. No external assistance was provided, to minimize interviewer bias. Baseline scores were recorded on the day of surgery, with follow-up data collected at 3, 6, and 12 months via hospital visits and questionnaires by email and phone calls. Adverse events, including type, severity, and duration, were reported at each time point. Primary outcomes focused on treatment efficacy and safety, while secondary outcomes examined the duration of effects, subgroup responses based on demographics and KL grade, and MFAT’s feasibility as a temporary alternative to arthroplasty for KL grade IV OA. Treatment failure was defined by treatment conversion to surgery (arthroplasty) or re-injection with MFAT, corticosteroids, PRP, or hyaluronic acid within the first 12 months of the follow-up period. The primary outcome of the study was the change in total KOOS score from baseline to 12 months, reflecting overall knee function. Secondary outcomes included changes in the KOOS subscales (Symptoms, Pain, ADL, Sport/Rec, and QoL) at 3, 6, and 12 months, subgroup differences according to sex, BMI, and KL grade, as well as the incidence and nature of adverse events.

### 2.4. Statistical Analysis

We performed all analyses using R version 4.4.2. Descriptive statistics, including counts, percentages, means, standard deviations, and ranges, were calculated for all covariates and outcome variables to provide an overview of the dataset. This initial exploration allowed us to identify key characteristics of the data and inform subsequent modeling decisions.

We first conducted exploratory analyses, including individual profile plots and mean progression plots for KOOS and its subscales (Symptoms, Pain, ADL, Sport and Recreation, and QoL). These visual inspections revealed consistent patterns across outcomes.

To determine the final model, a linear mixed-effects model was performed with KOOS as the primary outcome variable. The analysis began by identifying the need for splines and determining optimal knot placement based on visual inspection and model fit, assessed using Akaike’s Information Criterion (AIC), a measure of model quality that balances fit and complexity. Subsequently, the inclusion of random intercepts and slopes was evaluated using likelihood ratio tests. The next step involved selecting the most appropriate covariance structure to best represent the data. Covariates, including sex, BMI, KL grade, and age, as well as their interactions with time, were then assessed, and only those with significant effects (*p* < 0.05) were retained in the final model.

Once the final model was established for KOOS, this structure was replicated for each subscale. This approach was chosen to leverage the observed consistency in patterns across outcomes while maintaining focus on KOOS as the primary variable. Additionally, this strategy facilitates easier comparison between the models, as the same structure is applied across all subscales, ensuring consistency in the analytical framework. Furthermore, post hoc testing was performed for each subscale to assess differences between time points, providing a detailed understanding of the progression and significance of changes over time for each outcome.

## 3. Results

### 3.1. Patient Demographics

**Figure 1:**
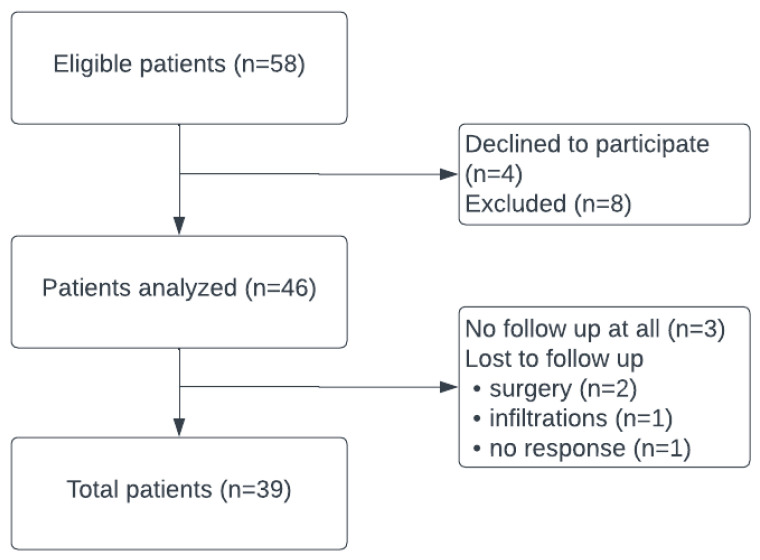
Participant selection process.

A total of 58 patients with OA were treated with MFAT infiltration. After applying the inclusion and exclusion criteria, 46 were eligible for further analysis. Fifteen percent (*n* = 7) of the study population was lost during follow-up. From 3 patients, we had no follow-up at all after the MFAT infiltration was performed. The other reasons were as follows: one patient was no longer reachable after the first visit at 3 months, one patient had a PRP injection four months after the MFAT infiltration for preventive reasons and therefore we excluded the patient, 2 patients had a conversion to surgery 3 to 6 months after the infiltration, thus treatment failure. Of those patients lost during follow-up, one patient had KL grade II, 3 patients had KL grade III, and 3 patients had KL grade IV. Because of the small numbers, these results were not further analyzed for selection bias. Figure 1 states the participant selection process.

The cohort consisted of 39 participants (Table 1), with a balanced representation of males (56.41%) and females (43.59%). The mean age was 55.51 years (SD = 10.65; range: 29–70 years). BMI distribution revealed no cases of underweight, with 38.46% classified as normal weight, 48.72% as overweight, and 10.26% as obese. For further analysis, the categories of overweight and obese were combined. Additionally, the KL grade assessment showed that 28.20% of participants had grade II OA, 35.90% had grade III OA, and 35.90% had grade IV OA.

### 3.2. KOOS (Sub)Scales

Table 2 presents the mean scores and standard deviations for the KOOS scale and its subscales across the four time points. The results indicate an overall increase in scores up to 6 months, followed by a slight decrease to 12 months. However, the scores at 12 months remain higher than those at baseline, reflecting an improvement in knee problems over time. Notably, all (sub)scales, except for the Symptoms subscale, showed a difference of more than 10 points between baseline and 12 months, which is an accepted minimal clinically important difference (MCID) [21].

**Figure 2:**
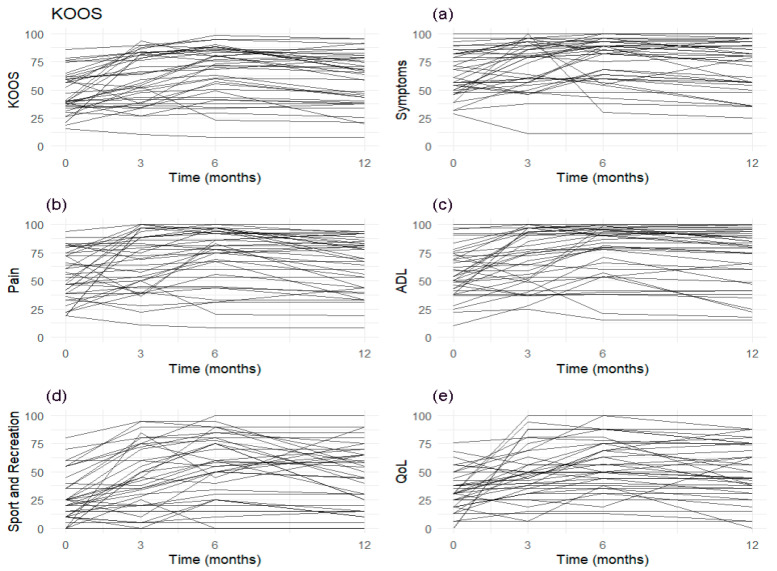
Profile plots for the Knee injury and Osteoarthritis Outcome Score (KOOS) scale and its subscales: (**a**) Symptoms, (**b**) Pain, (**c**) Activities of Daily Living (ADL), (**d**) Sport and Recreation, and (**e**) Quality of Life (QoL).

The profile plots for the total KOOS scale and its (sub)scales (Figure 2) reveal substantial variability in both the initial scores and their trajectories over time. This variability is consistently observed across all subscales, indicating individual differences in both baseline values and the evolution of scores. This variability was already expected, given the high standard deviations reported in Table 2, highlighting substantial heterogeneity in the sample.

**Figure 3:**
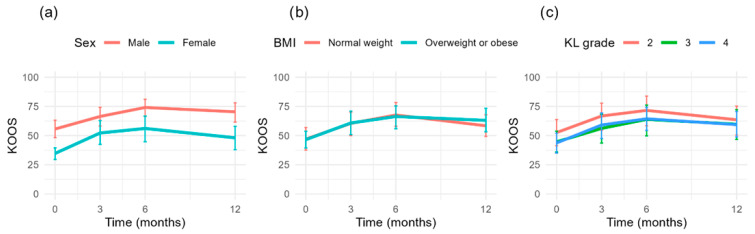
Mean KOOS scores over time, stratified by (**a**) Sex, (**b**) Body Mass Index (BMI), and (**c**) Kellgren-Lawrence (KL) grade.

**Figure 4:**
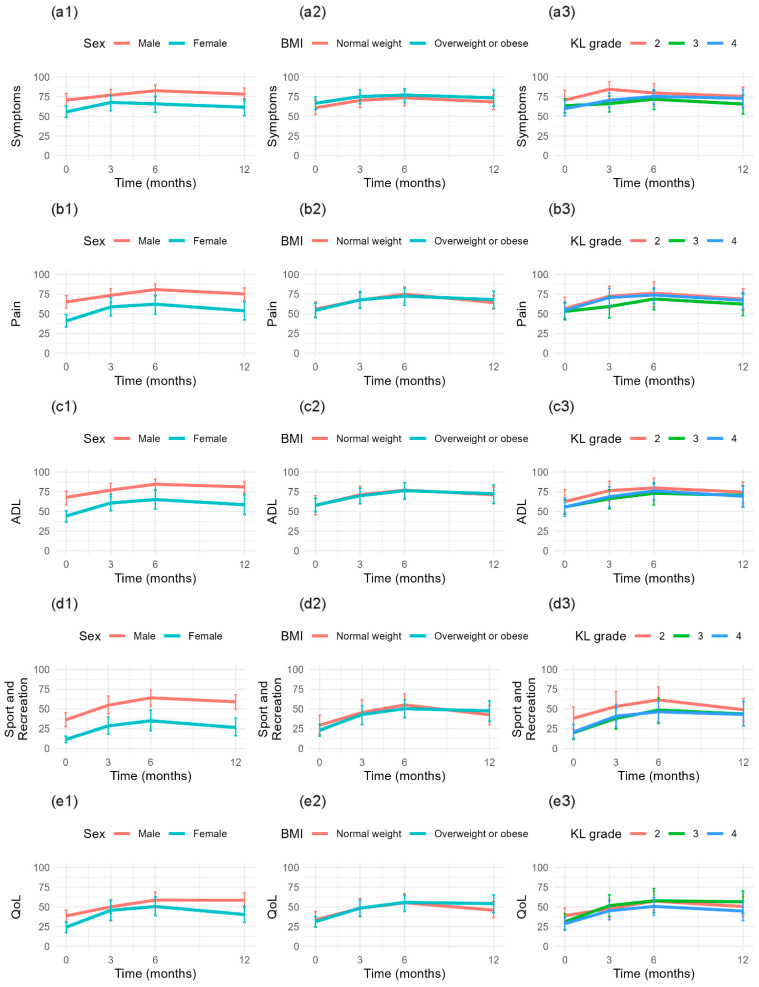
Mean scores for KOOS subscales (**a**) Symptoms, (**b**) Pain, (**c**) ADL, (**d**) Sports and Recreations, and (**e**) QoL; stratified by (**1**) Sex, (**2**) BMI, and (**3**) KL grade.

The mean plots (Figure 3) illustrate that the progression of KOOS scores is non-linear, with an initial increase up to 6 months followed by a decline. A clear distinction is observed between males and females, with males consistently scoring higher at all time points. This pattern is also evident across all subscales (Figure 4). For BMI categories, the mean trajectories largely overlap, showing minimal differences between groups. Similarly, for KL grade, differences between groups are limited, particularly between grades III and IV, which appear to follow a nearly identical trajectory over time.

### 3.3. Model Development and Selection

When determining the optimal number of splines for the time variable, the model with the lowest AIC included two knots at month 3 and month 6. Based on visual inspection of the individual profile plots, we assessed the need for random intercepts and slopes. However, due to convergence issues likely related to the small sample size, random slopes could not be incorporated, and we proceeded with random intercepts only. The covariance structure with the lowest AIC was the unstructured model, which was selected as the final covariance structure.

In evaluating covariates, the model with the lowest AIC included sex assigned at birth, BMI, and KL grade, while age did not improve the model and was excluded. Among these, only sex had a statistically significant effect (*p* < 0.05). The final model (Table 3) was therefore based on sex as the sole significant covariate. According to the model, females had significantly lower KOOS scores at all time points compared to males.

All splines were significant, indicating nonlinear changes over time. Post hoc testing showed that all time points were significantly different from one another, except for months 3 and 12. Scores at 12 months were significantly higher than at baseline, reflecting improved outcomes over time.

**Figure 5:**
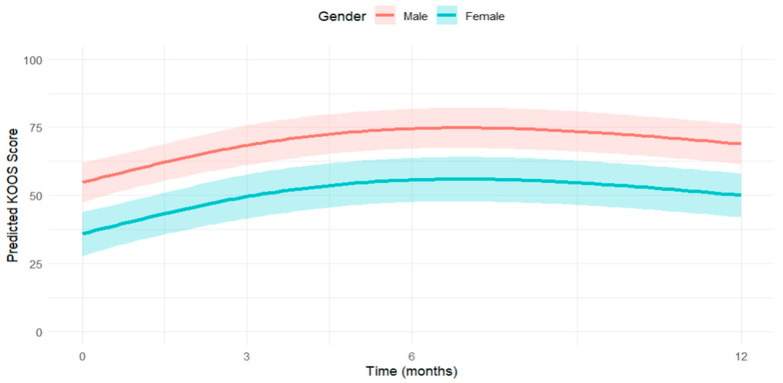
Predicted KOOS scores over time by sex assigned at birth.

**Figure 6:**
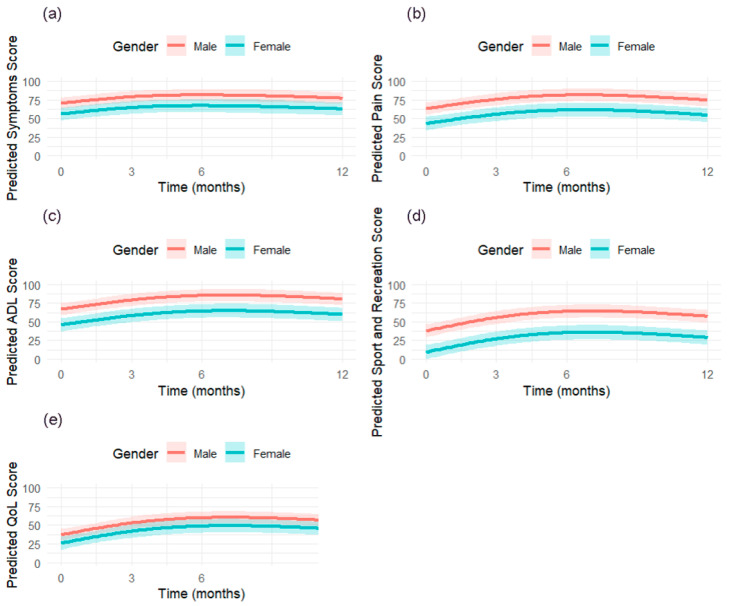
Predicted subscale scores over time by sex for (**a**) Symptoms, (**b**) Pain, (**c**) Activities of Daily Living (ADL), (**d**) Sport and Recreation, and (**e**) Quality of Life (QoL).

The evolution of KOOS scores over time for males and females is visually depicted in Figure 5, showcasing the upward trend until 6 months, followed by a slight decline at 12 months, with males consistently scoring higher than females. The figure also includes confidence intervals, providing a visual representation of the variability in predicted scores. The difference in the predicted subscale scores over time by sex is represented in Figure 6.

In Table 4, we present the results of the final mixed-effects models for the KOOS subscales, including Symptoms, Pain, Activities of Daily Living (ADL), Sport and Recreation, and Quality of Life (QoL). Across all subscales, females consistently scored significantly lower than males, indicating worse knee problems.

All spline terms were significant in each subscale, except for certain cases. Specifically, in the Symptoms subscale, the first spline (0–3 months) and the third spline (6–12 months) were not significant. Similarly, in the Pain subscale, the third spline (6–12 months) was also not significant.

Post hoc testing revealed no significant differences between the scores at 3 months and 12 months across all subscales, aligning with the results for the total KOOS score. However, a significant difference was consistently observed between baseline (0 months) and 12 months, indicating improved scores after one year for each subscale. Further analysis revealed that, for the Symptoms subscale, there was no significant difference between 3 and 6 months. For the QoL subscale, no significant difference was found between 6 and 12 months, indicating a plateau in knee-related quality of life after six months.

### 3.4. Adverse Events

During the 12 months of follow-up, 18% (n = 7) of the patients experienced synovitis, which lasted a maximum of 2 months. These symptoms of pain and swelling all resolved spontaneously without further intervention. One patient suffered postoperative nausea for 2 days, a well-known side effect of general anesthesia. However, no severe adverse effects were noted.

## 4. Discussion

The main finding of this study was a significant improvement in KOOS scores and all subscales at 12 months compared to baseline. Except for the subscale Symptoms, all improvements between baseline and 12 months exceeded the minimal clinically important difference of 10 points, confirming the clinical relevance of MFAT injections [14,18,21]. These findings are consistent with previous research, highlighting the potential of single-dose MFAT infiltration as a promising treatment for knee OA [3,6,8,9,10,11,12,13,14,15,16,17,18,24,25,26,27,28]. A systematic review by Migliorini et al. confirmed significant improvements across all assessed clinical and functional outcomes [29]. The KOOS Symptoms subscale was the only domain without a clinically relevant improvement in this study. This may be because it measures stiffness, swelling, and grinding issues linked to structural damage and biomechanics, which are less responsive to MFAT’s effects. Subjective variability in symptom reporting and the need for long-term tissue remodeling might also explain the slower improvement, as noted by Lapuente et al. [30]. Another contributing factor could be the KOOS Symptoms subscale itself, which includes fewer items and may therefore be less sensitive to capture clinically relevant improvements compared with other subscales.

The trajectory of KOOS scores over time revealed a nonlinear trend. Most subscales showed substantial improvement up to 6 months, followed by a slight decline in improvement, except for QoL, where a plateau was reached after 6 months. Between 6 and 12 months, the total KOOS score declined by 5.6 points (−8.4% relative to the 6-month value). Despite this reduction, scores remained 14.1 points higher (+30%) compared with baseline, underscoring that the overall clinical benefit was sustained over one year. Several studies have shown a decrease in treatment effectiveness over time, but the timing of this decline varies. This pattern may reflect the interplay of physiological and behavioral changes during recovery. The initial rapid improvement is attributed to the acute effects of MFAT infiltration, including reduction of joint inflammation, enhanced lubrication, and early functional restoration driven by pain relief. However, as the recovery progresses, the body’s capacity for tissue remodeling and regeneration might plateau, limiting further physical improvements. Concurrently, behavioral factors such as declining adherence to rehabilitation protocols, reduced physical activity, or the psychological adjustment to perceived improvements could contribute to the observed deceleration. These dynamics underscore the complexity of the recovery trajectory and highlight the need for strategies to maintain gains beyond the initial recovery phase. Nevertheless, multiple studies have shown significant improvements up to 2 years of follow-up [3,5,6,15,17,25,28]. These studies also highlight that, although outcomes deteriorate after a certain period, improvements relative to baseline remain. To illustrate, Russo et al. reported improved outcomes up to 3 years after MFAT infiltration for knee OA [25]. In addition, Onorato et al. observed improved outcomes up to 4 years of follow-up in patients with early knee OA [17].

Sex differences in KOOS scores were a consistent finding, with females scoring significantly lower than males across all time points and subscales. Van Genechten et al. also found lower scores, particularly in the KOOS pain score in females [11]. However, male and female patients follow a similar trend. This is confirmed in the literature, where most research could not prove a significant difference in the improvement of outcomes between sexes [3,17,30]. The largest disparities were observed in the Sport and Recreation subscale, where female participants approached a score of 0 (the worst outcome). This could be attributed to biological factors, such as hormonal influences on ligament and muscle function, or psychosocial differences, including variations in activity levels, coping strategies, and pain perception between sexes. Conversely, the near absence of sex differences in the QoL subscale suggests that both sexes perceive broader improvements in overall life quality similarly. Importantly, baseline KOOS scores were descriptively lower in females, suggesting that sex differences in outcomes may partly reflect pre-existing disparities rather than differential treatment response. Literature has shown that the prevalence of knee OA is higher among females [19,29]. Moreover, females are diagnosed with more severe knee OA and experience more debilitating pain compared to males [31,32]. However, the baseline difference was not statistically significant in our sample.

Previous research suggests that patients with lower KL grades may benefit more from MFAT injection than patients with advanced OA. Babowski et al. found that only patients with KL grade II OA significantly improved, while the scores of patients with KL grade IV deteriorated for every outcome measure [18]. However, only seven patients with KL grade IV were included in this study. Lapuente et al. found a more remarkable improvement in KL grade III compared to grade IV [30]. Similarly, the findings of Screpis et al. and Migliorini et al. revealed smaller improvements in outcomes for more advanced OA [15,33]. On the contrary, this study found no significant differences when comparing different KL grades. This finding is particularly interesting, as many other studies tend to exclude patients with grade IV knee OA. Our study suggests that MFAT infiltration may still be effective in advanced OA and is supported by previously published data [19,24,27]. However, this interpretation should be made cautiously, given the small number of patients in this subgroup and the limited statistical power to detect differences. Nonetheless, patients with grade II exhibited better overall scores than those with grades III and IV, although the latter two showed nearly identical improvements.

In this sample, normal-weight and overweight or obese individuals followed nearly identical trajectories, indicating that BMI may not significantly influence treatment response. This is in line with the findings in prior research, where no other study could prove the deterioration of outcomes in obese people [12,13,17]. Larger studies are needed to clarify whether BMI truly has no impact on MFAT outcomes, or whether our analysis lacked sufficient power to detect subtle effects.

In our study, a minority of the patients experienced side effects, with synovitis being the most common. These side effects were minor, generally self-limiting, and did not require significant medical intervention. In this sample, 18% of the patients experienced synovitis after injection, of which all resolved spontaneously. However, the occurrence of synovitis varies widely in previous research, and was not mentioned in most studies [10,11,15,17,18,25]. Injection-related factors, including the total volume administered, might play a role in this variability, as larger boluses can temporarily increase intra-articular pressure and provoke synovitis. Technical aspects such as the harvesting site, the processing of adipose tissue, and the injection technique (e.g., intra-articular placement accuracy) could influence the risk of synovial irritation. Future studies should evaluate these determinants to better understand variability in adverse event rates and to optimize the safety profile of MFAT treatment.

The strengths of this study include its longitudinal design, which allowed for the evaluation of both short- and long-term effects of MFAT infiltration on KOOS scores and subscales. Including patients with grade IV knee OA represents a real-life situation, as we experience a high demand for less-invasive therapies among these patients. However, one year of follow-up is a limitation, as previous studies have shown effects lasting up to several years after infiltration.

The small and heterogeneous sample size limited the study’s ability to detect more nuanced effects and explore complex random effects structures, such as random slopes. This sample size impacts the generalizability of our findings, as does the potential for bias introduced by loss to follow-up. Additionally, relying on self-reported KOOS scores introduces inherent biases, including subjectivity and recall errors, which could affect the accuracy of the reported improvements. Larger cohorts are needed to confirm these findings and to better capture smaller but clinically relevant differences.

Another important limitation of our study is the absence of a comparator group (e.g., PRP, corticosteroid injections, or sham). Therefore, improvements cannot be attributed solely to the MFAT intervention. Placebo effects are well-documented in intra-articular injection studies and may have contributed to the observed benefits, as the procedure itself can induce symptomatic relief through patient expectation or transient changes in joint homeostasis [34]. In addition, regression-to-the-mean must be considered, as patients often present during symptom exacerbation, and a spontaneous reduction of symptoms toward their long-term average could partially explain the improvements seen over time. Future randomized controlled trials with appropriate control arms are needed to disentangle the specific therapeutic effect of MFAT from these non-specific influences [35].

Nevertheless, the substantial improvements remain undeniable, and this study confirmed the effectiveness and safety of MFAT injection therapy. Future studies with larger cohorts are essential to validate these findings and assess the clinical implications of nonlinear progression and eventual plateau in KOOS scores. To minimize biases, randomized controlled trials are necessary to compare MFAT injections with PRP and other injection therapies. The decline in efficacy after a certain period raises important questions about the potential need for reinjections or combination therapies. Studies with longer follow-up are needed to confirm the duration of effect and precise the timing strategy for re-injection. Furthermore, Koh et al. demonstrated significant dose-dependent improvements in pain and function following a single MFAT injection [28]. Therefore, future research should also prioritize determining the optimal dosage for treatment.

## 5. Conclusions

This study found that micro-fragmented adipose tissue (MFAT) injections significantly improve knee function and quality of life in OA patients, with effects lasting at least 12 months after infiltration. Peak benefits at six months slightly declined by 12 months, indicating the need for strategies to sustain long-term outcomes. Significant improvement in KOOS scores and all subscales at 12 months compared to baseline was demonstrated for KL grades II-IV. Minor, self-limiting side effects, such as synovitis, confirmed MFAT’s favorable safety profile.

Clinically, this study highlights the potential utility of MFAT infiltration as a treatment option for patients across all KL grades, including those with advanced OA. MFAT shows promise as a minimally invasive option to delay or avoid surgery, with further research needed to optimize reinjection protocols and patient selection.

## Figures and Tables

**Table 1:** Patient characteristics.

Characteristic	Category	*n* (%)
Sex assigned at birth	Male	22 (56.41)
	Female	17 (43.59)
Body Mass Index	Underweight	0 (0.00)
	Normal	15 (38.46)
	Overweight	19 (48.72)
	Obese	4 (10.26)
	Missing	1 (2.56)
Kellgren-Lawrence	Grade 2	11 (28.20%)
	Grade 3	14 (35.90%)
	Grade 4	14 (35.90%)

**Table 2:** Mean KOOS scores and subscale scores across time points.

	Baseline	3 Months	6 Months	12 Months
Outcome Variables	Mean (SD)	Mean (SD)	Mean (SD)	Mean (SD)
KOOS	46.54 (18.08)	60.24 (22.20)	66.27 (22.80)	60.68 (23.08)
Symptoms	64.05 (19.38)	72.79 (20.89)	75.20 (21.53)	70.92 (23.41)
Pain	54.54 (21.98)	67.00 (25.04)	72.67 (24.70)	65.85 (24.10)
ADL	57.61 (22.02)	70.00 (23.98)	76.10 (23.83)	71.10 (25.59)
Sport and Recreation	25.38 (21.16)	43.31 (29.34)	51.41 (29.96)	44.87 (28.65)
QoL	32.31 (18.37)	48.10 (23.80)	55.0 (24.47)	50.52 (25.34)

Note: Each (sub)scale ranges from 0 to 100, with 0 indicating extreme knee problems and 100 indicating no knee problems, with a MCID of 10. Abbreviations: KOOS = Knee injury and Osteoarthritis Outcome Score, ADL = Activities of Daily Living, QoL = Quality of Life, SD = Standard Deviation.

**Table 3:** Results of the final mixed-effects model for KOOS Scores.

Predictor	B	SE	t	*p*	95% CI
(Intercept)	54.76	3.72	14.73	<0.001	47.40; 62.12
Spline 1 (0–3 months)	17.56	3.33	5.27	<0.001	10.96; 24.17
Spline 2 (3–6 months)	30.70	5.84	5.26	<0.001	19.13;42.26
Spline 3 (6–12 months)	6.28	2.11	2.98	0.004	2.10; 10.47
Sex (Female)	−18.85	5.04	−3.74	0.001	−29.07; −8.64

Abbreviations: B = Unstandardized coefficient, SE = Standard Error, t = t-value, *p* = *p*-value, CI = Confidence Interval.

**Table 4:** Results of the final mixed-effects models for the KOOS subscales.

	Predictor	B	SE	t	*p*	95% CI
Symptoms	(Intercept)	70.32	3.93	17.88	<0.001	62.53; 78.11
	Spline 1 (0–3 months)	8.67	5.01	1.73	0.086	−1.25; 18.60
	Spline 2 (3–6 months)	17.71	5.04	3.52	0.001	7.73;27.68
	Spline 3 (6–12 months)	1.64	2.81	0.58	0.561	−3.93; 7.21
	Sex assigned at birth (Female)	−14.37	5.38	−2.67	0.011	−25.27; −3.48
Pain	(Intercept)	63.26	4.23	14.96	<0.001	54.88; 71.63
	Spline 1 (0–3 months)	16.54	3.76	4.40	<0.001	9.09; 23.98
	Spline 2 (3–6 months)	27.12	7.17	3.78	<0.001	12.92; 41.33
	Spline 3 (6–12 months)	4.21	2.38	1.77	0.079	−0.50; 8.91
	Sex assigned at birth (Female)	−20.00	5.63	−3.55	0.001	−31.42; −8.58
ADL	(Intercept)	66.68	4.14	16.11	<0.001	58.48; 74.88
	Spline 1 (0–3 months)	17.04	4.30	3.96	<0.001	8.52; 25.55
	Spline 2 (3–6 months)	28.57	6.14	4.65	<0.001	16.40; 40.74
	Spline 3 (6–12 months)	6.69	2.39	2.80	0.006	1.95; 11.42
	Sex assigned at birth (Female)	−20.79	5.61	−3.71	0.001	−32.16; −9.43
Sport and Recreation	(Intercept)	37.80	4.42	8.55	<0.001	29.04; 46.56
	Spline 1 (0–3 months)	23.27	4.61	5.05	<0.001	14.14; 32.40
	Spline 2 (3–6 months)	40.83	6.91	5.91	<0.001	27.14; 54.51
	Spline 3 (6–12 months)	9.23	3.28	2.81	0.006	2.73; 15.73
	Sex assigned at birth (Female)	−28.48	5.80	−4.91	<0.001	−40.23; −16.73
QoL	(Intercept)	37.14	4.15	8.94	<0.001	28.91; 45.38
	Spline 1 (0–3 months)	19.89	3.53	5.64	<0.001	12.90; 26.88
	Spline 2 (3–6 months)	36.45	8.06	4.52	<0.001	20.49; 52.41
	Spline 3 (6–12 months)	9.12	2.48	3.67	<0.001	4.20; 14.03
	Sex assigned at birth (Female)	−11.10	5.34	−2.08	0.045	−21.92; −0.28

Abbreviations: KOOS = Knee injury and Osteoarthritis Outcome Score; ADL = Activities of Daily Living; QoL = Quality of Life; B = Unstandardized coefficient; SE = Standard Error; t = t-value; *p* = *p*-value; CI = Confidence Interval.

## Data Availability

The data presented in this study are available on request from the corresponding author due to privacy policy.

## Abbreviations

OA	Osteoarthritis
KOOS	Knee injury and Osteoarthritis Outcome Score
KL	Kellgren-Lawrence
MFAT	Micro-fragmented adipose tissue
NSAID	Non-steroidal anti-inflammatory drugs
MRI	Magnetic resonance imaging
QoL	Quality of Life
ADL	Activities of Daily Living
MSC	Mesenchymal stem cells
BMI	Body Mass Index
MCID	Minimal clinically important difference
